# Differential Regulation of Anthocyanins in Green and Purple Turnips Revealed by Combined De Novo Transcriptome and Metabolome Analysis

**DOI:** 10.3390/ijms20184387

**Published:** 2019-09-06

**Authors:** Hongmei Zhuang, Qian Lou, Huifang Liu, Hongwei Han, Qiang Wang, Zhonghua Tang, Yanming Ma, Hao Wang

**Affiliations:** 1 Institute of Horticultural Crops, Xinjiang Academy of Agricultural Sciences, Urumqi 830091, China (H.Z.) (H.L.) (H.H.) (Q.W.); 2 College of Horticulture, Northwest A & F University, Yangling 712100, China; 3 Key Laboratory of Plant Ecology, Northeast Forestry University, Harbin 150040, China; 4 Institute of Genetic Resources, Xinjiang Academy of Agricultural Science, Urumqi 830091, China

**Keywords:** pigment, turnip, gene expression, antioxidant, nutritional quality

## Abstract

Purple turnip *Brassica rapa* ssp. rapa is highly appreciated by consumers but the metabolites and molecular mechanisms underlying the root skin pigmentation remain open to study. Herein, we analyzed the anthocyanin composition in purple turnip (PT) and green turnip (GT) at five developmental stages. A total of 21 anthocyanins were detected and classified into the six major anthocynanin aglycones. Distinctly, PT contains 20 times higher levels of anthocyanins than GT, which explain the difference in the root skin pigmentation. We further sequenced the transcriptomes and analyzed the differentially expressed genes between the two turnips. We found that PT essentially diverts dihydroflavonols to the biosynthesis of anthocyanins over flavonols biosynthesis by strongly down-regulating one flavonol synthase gene, while strikingly up-regulating dihydroflavonol 4-reductase (DFR), anthocyanidin synthase and UDP-glucose: flavonoid-3-*O*-glucosyltransferase genes as compared to GT. Moreover, a nonsense mutation identified in the coding sequence of the DFR gene may lead to a nonfunctional protein, adding another hurdle to the accumulation of anthocyanin in GT. We also uncovered several key members of MYB, bHLH and WRKY families as the putative main drivers of transcriptional changes between the two turnips. Overall, this study provides new tools for modifying anthocyanin content and improving turnip nutritional quality.

## 1. Introduction

Turnip (*Brassica rapa* ssp. rapa), belongs to the Cruciferae family and represents one of the most important leaf and root vegetable crops for human consumption and animal fodder in China and throughout East Asia. Turnip vegetables provide dietary fiber, vitamin C, high amounts of glucosinolates [1,2,3], and are also an important source of dietary phenolic and other bioactive compounds [4,5]. There are several turnip varieties with purple colored root skin, which are highly appreciated by consumers. Similar to turnip, there are various *B. rapa* subspecies enriched with purple pigments previously characterized as anthocyanins [6].

Anthocyanins are secondary metabolites with health-promoting virtues, such as anti-oxidation, anti-mutation, prevention of cardiovascular disease, liver protection, and inhibiting the metastasis of tumor cells [7,8,9,10,11,12]. Besides, they also play important fundamental physiological functions in plants, including UV protection, pigmentation of flowers and fruits to attract pollinators and for seed dispersal, and responses to biotic and environmental stresses [13,14,15,16,17,18,19,20]. Therefore, the identification, analysis and genetic manipulation of anthocyanin metabolites have become an important topic in plant secondary metabolite research [21].

The biosynthesis and accumulation of anthocyanins are determined by metabolic networks correlated with the expression of several genes and regulatory factors [22]. During the past decades, extensive studies have been conducted to elucidate the biosynthetic pathway of anthocyanins in plants. Progressively, it has become evident that the anthocyanin biosynthetic pathway is a very well conserved network in plant species [23]. It starts with the chalcone synthase (CHS) mediated synthesis of naringenin chalcone from 4-coumaroyl-CoA and malonyl-CoA. Then, naringenin chalcone is isomerized by chalcone isomerase (CHI) to naringenin. Flavanone 3-hydroxylase (F3H) converts naringenin into dihydrokaempferol which can be further hydroxylated by flavonoid 3′-hydroxylase (F3′H) or flavonoid 3′,5′-hydroxylase (F3′5′H) into two other dihydroflavonols, dihydroquercetin and dihydrotricetin, respectively. Then, the three dihydroflavonols are converted into colorless leucoanthocyanidins by dihydroflavonol 4-reductase (DFR) and subsequently to colored anthocyanidins by anthocyanidin synthase (ANS). Anthocyanidins are glycolsylated to facilitate their accumulation in cells by the enzyme flavonoid 3-*O*-glucosyltransferase (UFGT) and might be further acylated with aromatic acyl groups by acyltransferases [18,22]. Although a well conserved biosynthetic pathway in plants, several studies have shown the species-specific peculiarity of anthocyanin regulation. For example, the numbers of structural genes (CHS, F3H, F3′H, CHI, DFR, ANS, UFGT, F3′5′H, etc.) vary considerably across species as do their expression levels [24,25,26,27]. Also, genetic mutations, microRNAs, transcription factors such as MYB, bHLH, WD40, WRKY, NAC, etc., have been linked to the regulation of anthocyanin biosynthetic structural genes through varying complex mechanisms among plants [14,26,27,28,29,30,31,32,33,34,35,36,37,38,39,40,41]. Therefore, in order to pinpoint the major players associated with quantitative and qualitative variations of anthocyanins in plant, a thorough investigation is necessary.

Herein, we investigated the anthocyanin compositions at different developmental stages in root skin of two turnip varieties (purple turnip and green turnip) widely grown in Xinjiang (China). In addition, we generated extensive transcriptome data and profiled the key genes involved in the differential pigmentation. The goal of this work was to elucidate the molecular and metabolic mechanisms underlying the differential pigmentation in turnips, as a foundation for the development of turnip varieties that are rich in anthocyanin compounds to meet the increasing demand for health-promoting components in our daily diet.

## 2. Results

### 2.1. De Novo Transcriptome Assembly and Gene Expression Profiles in the Two Turnips at Five Developmental Stages

**Figure 1:**
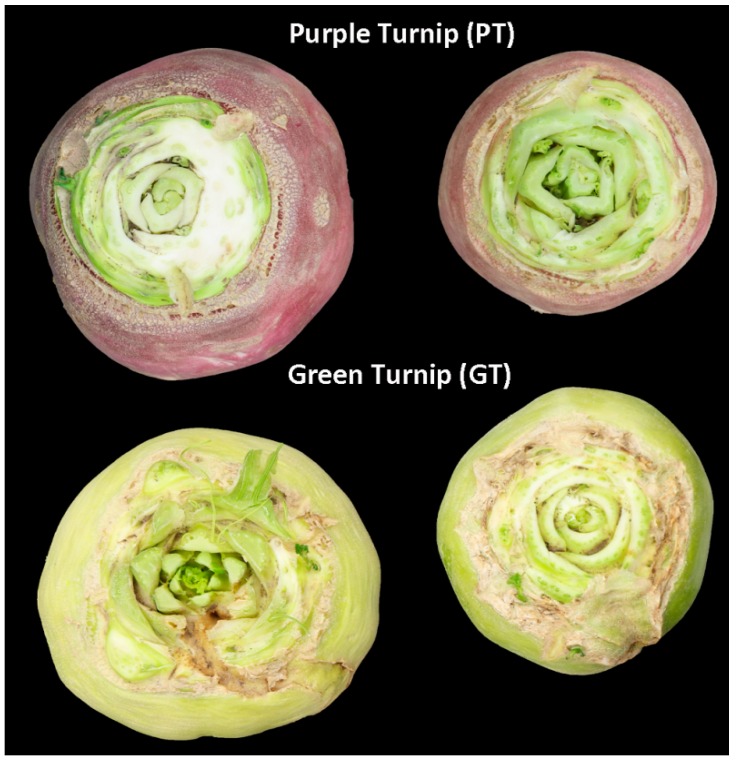
The phenotypes of young and mature purple-colored turnip and green-colored turnip roots.

In this work, two widely grown *Brassica rapa* ssp. rapa varieties in Xinjiang (China) including GT with green-colored root skin and PT with purple-colored root skin were studied. Skin samples were collected at five different developmental stages, namely seedling stage (S1, 15 days after sowing (DAS)), early stage of fleshy root expansion (S2, 30 DAS), full expansion stage of fleshy root (S3, 45 DAS), maturity stage of fleshy root (S4, 55 DAS) and harvest stage of fleshy root (S5, 65 DAS). The phenotypes of young and mature turnip roots for GT and PT are presented in Figure 1. The objective of the work was to elucidate the mechanisms underlying the differential skin coloration in these turnips with respect to the accumulation of anthocyanin compounds. First, we de novo sequenced and assembled the transcriptome from 30 samples of the two turnips.

**Figure 2:**
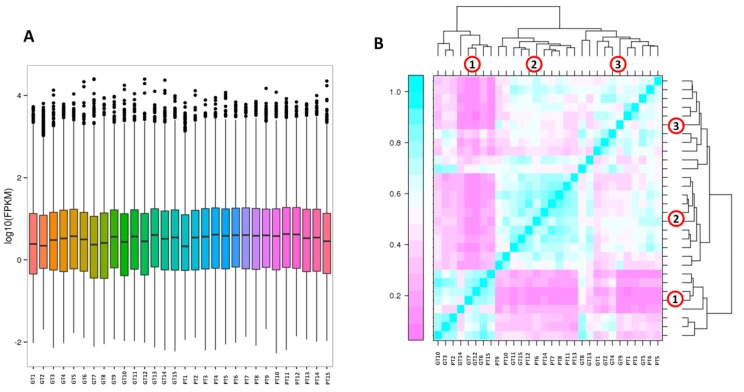
Overview of the transcriptome sequencing. (**A**) Gene expression profiles in the 30 libraries. PT represents the purple turnip while GT represents the green turnip; (**B**) heatmap clustering showing correlation among turnip samples based on global expression profiles. The number 1, 2 and 3 represent the 3 Clusters of samples.

The RNA-seq yielded a total of 232.22 Gb clean data, on average 6.20Gb for each sample with 90.74% of bases scoring Q30 and above (Table 1). A total of 76,152 unigenes were obtained after assembly using the Trinity software and 17,594 unigenes have length of more than 1 kb. The N50 length obtained was approximately 1443 bp (Table 2). The detected gene number in this study was much higher than the reported gene number (41,174 genes) in *Brassica rapa* ssp. pekinensis variety Chiifu-401-42 [42] or (40,708 genes) in *Brassica rapa* ssp. rapa [43]. We performed the functional annotation of the unigenes in various database, including NR, Swiss-Prot, KEGG, COG, KOG, GO and Pfam databases, which resulted in 52,449 unigenes successfully annotated (Table 3). The clean data of each sample was serialized with the assembled unigene libraries and the mapping result statistics are presented in Table S1. Gene expression levels were estimated with the fragments per kilobase of exon per million fragments mapped (FPKM) values ranging from 0.04 to 5,566,185 (Figure 2A).

Hierarchical clustering of the samples based on FPKM displayed 3 Clusters of samples. We did not observe a clear separation according to the developmental stages but to some extent, Clusters were related to the turnip variety. For example, Cluster 1 gathered mostly samples of GT, Cluster 2 grouped samples of PT, and Cluster 3 had two subgroups each mostly made of samples from a unique variety (Figure 2B). These results indicate that the global gene expression profile is quite uniform regardless of the developmental stages and only few differentially expressed genes between the two varieties may be associated with the difference in the turnip skin coloration.

### 2.2. Differentially Expressed Genes Between the Two Turnips and Analysis of Major Regulator Genes

**Figure 3:**
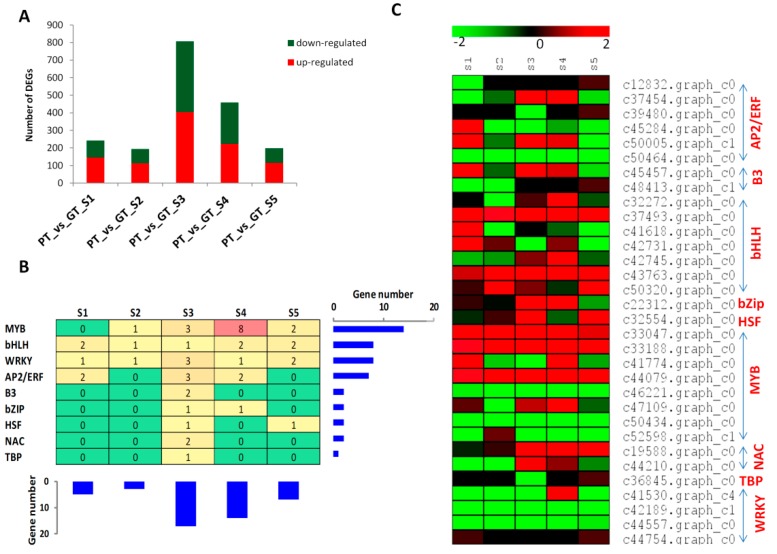
Transcription factors (TF) regulating the gene expression between the two turnips. (**A**) Number of up- and down-regulated genes between PT and GT at different developmental stages S1-S5. PT represents the purple turnip while GT represents the green turnip; (**B**) overview of the enriched TF family. The combined histograms showed the number of occurrence of genes belonging to each TF family or at each developmental stage; (**C**) Heatmap displaying the expression fold change (Log2 fold change) between PT and GT for the gene encoding transcription factors.

To identify the differentially expressed genes (DEG) related to turnip skin coloration, we compared the FPKM values of each gene in PT to GT at the different developmental stages and retained DEGs with fold change > 2 and a false discovery rate (FDR) correction set at *p* < 0.01 [44]. We detected 242, 194, 807, 459 and 199 DEGs at S1, S2, S3, S4 and S5, respectively (Figure 3A). The marked change in gene expression between the two turnips observed at the S3, implies that S3 may represent a key stage for turnip skin coloration.

Transcription factors (TF) are the major regulators of gene expression profiles [45]. We therefore extended the study on the major transcription factor families differentially expressed between the two turnips. Our analysis showed that nine main TF families modulate the global gene expression levels among the two turnips (Figure 3B,C). In addition, the highest number of TF could be noticed at S3, which correlates well with the observed significant DEGs at this developmental stage (Figure 3A,B), showing that TFs are the main drivers of gene expression changes leading to the differential turnip skin coloration. Among the detected TF families, MYB, bHLH and WRKY families showed more active members involved in gene regulation (Figure 3B,C), therefore we deduce that these TF families may be crucial for the regulation of structural genes involved in turnip skin coloration. Distinctly, the genes *c42189.graph_c1* (WRKY) was strongly down-regulated over most of the developmental stages in PT while the genes *c33188.graph_c0* (MYB), *c44079.graph_c0* (MYB) and *c37493.graph_c0* (bHLH) exhibited the opposite trend. Given the role of WRKY, MYB and bHLH TFs in the regulation of structural genes involved in pigment (flavonoid-anthocyanin) biosynthesis in plants [33], it is tempting to speculate that these four key genes are the major regulators during turnip skin color formation.

### 2.3. Detection of Anthocyanin Compounds in the Two Turnips

**Figure 4:**
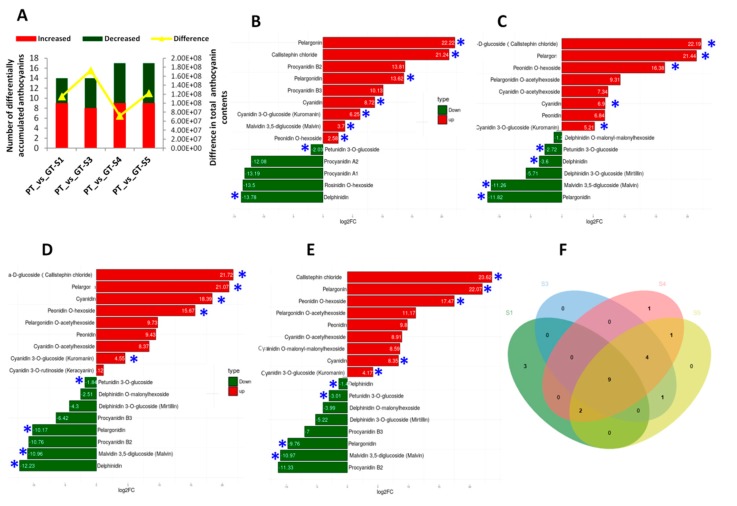
Temporal changes of anthocyanin content in two turnips. (**A**) Number of increased and decreased anthocyanins between PT and GT at different developmental stages S1–S5. The yellow line shows the difference in total anthocyanin content between PT and GT. PT represents the purple turnip while GT represents the green turnip; (**B**–**E**) differentially accumulated anthocyanins at S1, S3, S4, and S5 and their log2 fold change values between PT and GT. The asterisks mark the metabolites constantly differentially accumulated at the 4 developmental stages between PT and GT. (**F**) Venn diagram depicting the number of shared and unique differential accumulated anthocyanins between PT and GT at the 4 developmental stages.

Anthocyanins are the most important flavonoid colorants in plants [46]. We detected and determined 18, 18, 18 and 19 diverse anthocyanins at S1, S3, S4 and S5, respectively, resulting in 21 unique anthocyanin compounds in skin of the two turnips using the targeted-metabolomics approach (Table 4, Table S2). It is worth mentioning that the skin samples collected at the S2 from GT were deteriorated, so have not been used for this analysis. We did not detect procyanidin A1 and procyanidin A2 in PT, while GT did contain four anthocyanins: pelargonin and pelargonidin 3-*O*-beta-D-glucoside, pelargonidin *O*-acetylhexoside and cyanidin *O*-acetylhexoside (Table 4). With the determined quantities of all anthocyanins combined together, PT skin contained 20 times higher level of anthocyanins as compared to GT (Table S2). Also, the highest difference in total anthocyanin content between PT and GT during the developmental stages was observed at S3 (Figure 4A), which further supports the premise that S3 is the key for stage for turnip skin coloration. We investigated the differential accumulated metabolites (DAM) between the two turnips based on the variable importance in projection (VIP) ≥ 1 and fold change ≥ 2 or fold change ≤ 0.5 [47]). A total of 14, 14, 17 and 17 DAMs were recorded at S1, S3, S4 and S5, respectively (Figure 4A–E), resulting in all the 21 unique anthocyanin compounds differentially accumulated at least at one developmental stage between the two turnips. In addition, nine anthocyanins were constantly differentially accumulated in skin of the two turnips at all the four developmental stages (Figure 4F, Table S3). For most of these anthocyanin compounds, they showed presence/absence patterns among the two turnips skins, indicating that they are the key components conferring the differential pigmentation.

### 2.4. Mapping of Differential Genes and Metabolites Related to Flavonoid-Anthocyanin Biosynthesis Pathway

**Figure 5:**
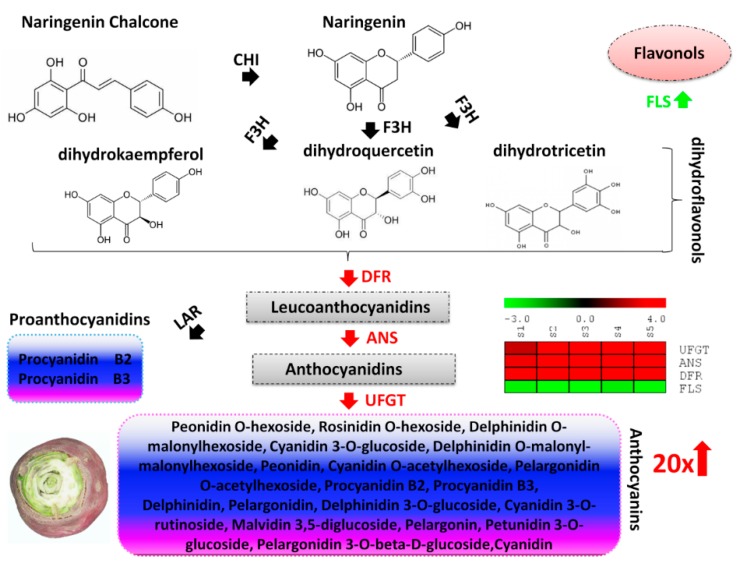
Proposed model of the molecular mechanism leading to the high anthocyanin content in the the purple turnip (PT). Naringenin chalcone is isomerized by chalcone isomerase (CHI) to naringenin. Flavanone 3-hydroxylase (F3H) converts naringenin into dihydroflavonols (dihydrokaempferol, dihydroquercetin or dihydrotricetin). Then, the three dihydroflavonols are converted into colorless leucoanthocyanidins by dihydroflavonol 4-reductase (DFR) and subsequently to colored anthocyanidins by anthocyanidin synthase (ANS). Anthocyanidins are glycolsylated to facilitate their accumulation in cells by the enzyme flavonoid 3-*O*-glucosyltransferase (UFGT). Proanthocyanidins are generated by the action of leucoanthocyanidin reductase (LAR) from leucoanthocyanidins. DFR, ANS and UFGT were found significantly up-regulated in PT leading to a high content of 17 anthocyanins compounds (more than 20 times compared to the green turnip). In contrast, FLS was found significantly down-regulated and may lead to a weak accumulation of flavonols. PT tends to prioritize anthocyanins accumulation by diverting dihydroflavonols to the anthocyanins biosynthesis pathway.

**Figure 6:**
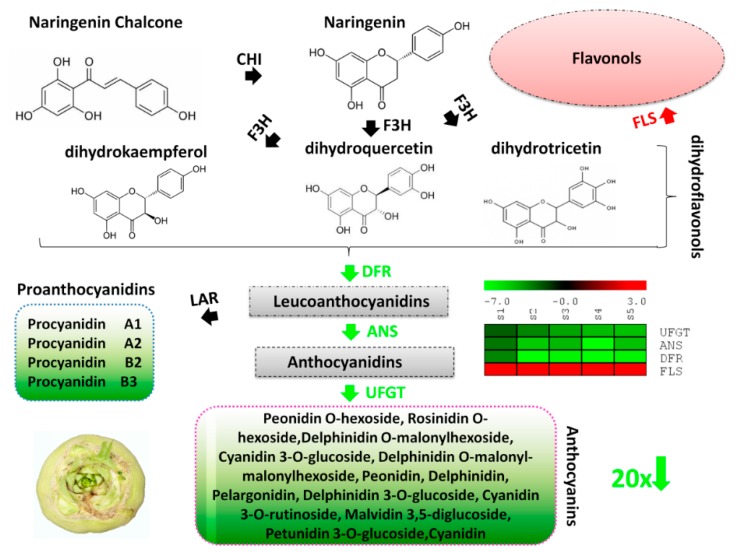
Proposed model of the mechanism leading to the low anthocyanin content in the green turnip (GT). Naringenin chalcone is isomerized by chalcone isomerase (CHI) to naringenin. Flavanone 3-hydroxylase (F3H) converts naringenin into dihydroflavonols (dihydrokaempferol, dihydroquercetin or dihydrotricetin). Then, the three dihydroflavonols are converted into colorless leucoanthocyanidins by dihydroflavonol 4-reductase (DFR) and subsequently to colored anthocyanidins by anthocyanidin synthase (ANS). Anthocyanidins are glycolsylated to facilitate their accumulation in cells by the enzyme flavonoid 3-*O*-glucosyltransferase (UFGT). Proanthocyanidins are generated by the action of leucoanthocyanidin reductase (LAR) from leucoanthocyanidins. DFR, ANS and UFGT were found significantly down-regulated in GT leading to a low content of 13 anthocyanins compounds (less than 20 times compared to the purple turnip). In contrast, FLS was found significantly up-regulated and may lead to a high accumulation of flavonols. PT tends to prioritize flavonol accumulation by diverting dihydroflavonols to the flavonols biosynthesis pathway.

The biosynthetic pathway of anthocyanins has been well-characterized in plants [22]. In order to predict the molecular mechanisms leading to the differential skin coloration in the two turnips, we have reconstructed the flavonoid-anthocyanin biosynthesis pathways (Figure 5 and Figure 6). First, we searched among the DEGs, those coding for enzymes involved in the flavonoid-anthocyanin biosynthesis pathways. We obtained four genes namely, flavonol synthase [EC:1.14.11.23] (*c43941.graph_c0*, FLS), dihydroflavonol-4-reductase [EC:1.1.1.234] (*c39842.graph_c0*, DFR), anthocyanidin synthase [EC:1.14.11.19] (*c45741.graph_c0*, ANS), and UDP-flavonoid glucosyl transferase [EC:2.4.1.91] (*c48211.graph_c0*, UFGT). All these enzymes mainly participate in the late steps of the flavonoid-anthocyanin biosynthesis pathways.

The flavonoid-anthocyanin biosynthesis pathways start from the key amino acid phenylalanine to produce 4-coumaroyl CoA by phenylalanine ammonia-lyase, cinnamic acid 4-hydroxylase and 4-coumarate CoA ligase [48]. The main precursors for flavonoids are 4-coumaroyl CoA and three molecules of malonyl CoA that produce chalcone by chalcone synthase (Dixon and Steele, 1999). Then, the pathway is catalyzed by a number of enzymes to yield flavanones (via chalcone isomerase), dihydroflavonols (via flavanone 3-hydroxylase) [49]. Dihydroflavonols are the keystone substrates for the biosynthesis of flavonols (via FLS) and anthocyanins (via DFR). In this study, we observed a constant down-regulation of one FLS in PT while a significant up-regulation of the expression level of one DFR during all the five developmental stages (Figure 5 and Figure 6), indicating that PT tends to prioritize the anthocyanins biosynthesis over flavonols. Next, leucoanthocyanidins which are generated from DFR are converted into anthocyanidins (via ANS) [48]. Similar to DFR, we noticed a stout up-regulation of one ANS throughout PT growth pointing to high accumulation of anthocyanidins (Figure 5 and Figure 6). Finally, anthocyanidins are converted into anthocyanins via UFGT [49]. We identified one UFGT significantly and constantly up-regulated in PT, showing a mechanism towards a strong accumulation of anthocyanins (Figure 5 and Figure 6). Based on the metabolite detection and quantification, we confirm that the accumulated anthocyanins conferring the purple pigmentation in PT are mainly peonidin *O*-hexoside, cyanidin 3-*O*-glucoside (kuromanin), pelargonidin, malvidin 3,5-diglucoside (calvin), pelargonin, pelargonidin 3-*O*-beta-D-glucoside (callistephin chloride) and cyanidin (Figure 5 and Figure 6). On the opposite, delphinidin and petunidin 3-*O*-glucoside are enriched in the skin of GT and may confer the greenish coloration (Figure 5 and Figure 6).

**Figure 7:**
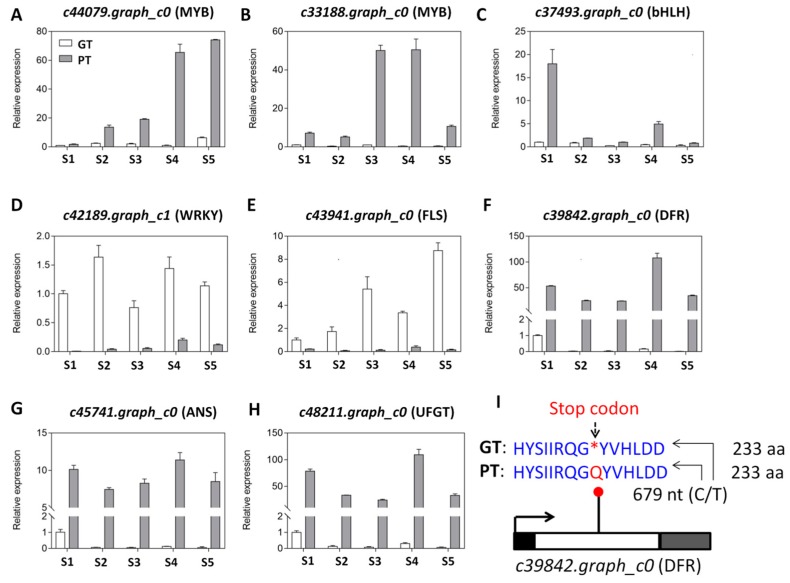
Quantitative real time PCR validation of selected candidate genes predicted to differentially affect the anthocyanin profiles in the two turnips. (**A–H**) Relative expression level of *c33188.graph_c0* (MYB), *c44079.graph_c0* (MYB), *c37493.graph_c0* (bHLH), *c42189.graph_c1* (WRKY), *c43941.graph_c0* (FLS), *c39842.graph_c0* (DFR), *c45741.graph_c0* (ANS) and *c48211.graph_c0* (UFGT) between PT and GT at five developmental stages (S1–S5). PT represents the purple turnip while GT represents the green turnip and are represented by the grey and white bars, respectively. The error bar represents the SD of biological replicates. The *Actin* gene was used as the internal reference gene for normalization; (**I**) identification of a non-sense mutation in the gene *c39842.graph_c0* (DFR) by comparing the sequences between PT and GT. The single nucleotide polymorphism (C/T) is located at the position 679 within the coding sequence of the gene and is predicted to generate an amino acid (aa) Q in PT while a stop codon in GT. The white box represents the exon while the black and gray boxes represent the UTR5′and UTR3′, respectively. The arrow indicates the transcription start site and transcription orientation.

To confirm the differential expression levels of the four candidate structural genes together with the four key transcription factors detected by the RNA-seq analysis, we performed a quantitative real-time PCR (Table S4). The results showed that the genes *c43941.graph_c0* (FLS) and *c42189.graph_c1* (WRKY) were obviously down-regulated over the developmental stages in PT while the genes c39842.graph_c0 (DFR), *c45741.graph_c0* (ANS), *c48211.graph_c0* (UFGT), *c33188.graph_c0* (MYB), *c44079.graph_c0* (MYB) and *c37493.graph_c0* (bHLH) were all found clearly up-regulated over the developmental stages in PT (Figure 7A–H). The qRT-PCR results were therefore in perfect concordance with the RNA-seq report.

### 2.5. Detection of SNPs within the Four Candidate Structural Genes Regulating the Differential Skin Coloration in Turnips

Differential gene expression among individuals is not only caused by the modulation of transcription factors but could result from variations in the nucleotide sequences [50]. Herein, we investigated the single nucleotide polymorphisms (SNP) within the sequences of the four differentially expressed candidate structural genes (*c43941.graph_c0*, *c39842.graph_c0*, *c45741.graph_c0* and *c48211.graph_c0*) associated with the anthocyanin biosynthesis pathways, which were predicted to modulate the pigment formation in turnip skin. Sequence comparison of the unigenes between the two turnips unveiled a putative SNP in *c39842.graph_c0* (DFR). In the DFR gene which has a single coding sequence (CDS) of 1,158 nucleotides (nt), a point-nonsense mutation (C/T) at the position 679 nt was detected in the CDS. The SNP has an allele depth (number of reads) of 0/51 for GT and 113,774/0 for PT. This indicates that the allele T which induces a stop codon in the resulting protein is likely to be present only in GT while the allele C is apparently only present in PT (Figure 7I).

## 3. Discussion

Anthocyanin containing fruits and vegetables are an integral part of human diet without any known adverse effect [51]. In this study, we profiled the anthocyanin composition of two widely grown turnips (*Brassica rapa* ssp. rapa) in Xinjiang (China), with purple- (PT) and green-colored (GT) skins (Figure 1) and investigated the underlying genetic basis. Common aglycones of anthocyanin are pelargonidin, cyanidin, delphinidin, peonidin, petunidin, and malvidin [52]. Using the ultra-performance liquid chromatography and tandem mass spectrometry technologies, we determined 17 anthocyanin compounds in the turnip skins, classified into the common six aglycones of anthocynanins (Table 4, Table S2). In addition, we also detected four proanthocyanidins compounds, including procyanidin A1, procyanidin A2, procyanidin B2 and procyanidin B3 (Table 4, Table S2). There were no formal studies on the anthocyanins composition in *Brassica rapa* ssp. rapa, however, according to previous studies, cyanin glycosides are the major anthocyanin substances accumulated in Brassica crops [53,54,55,56]. Nine cyanidin anthocyanins were detected in purple cauliflower and purple cabbage [53]. Li [54] analyzed anthocyanin extracts of ′violet′ purple cabbage and obtained eight different anthocyanin components, whose basic component is cyanidin-malonyl-glucoside. Later on, Guo et al. [55] examined purple seaweed sprouts, purple turnips, and purple cabbage and identified 23 anthocyanins compounds, composed of 17 cyanidins and six pelargonidin. Recently, Park et al. [56] also found 11 anthocyanins, predominantly cyanindin in purple Kohlrabi (*Brassica oleracea* var. gongylodes). We deduce that there is a large variation of the anthocyanin profiles among purple Brassica vegetables but *Brassica rapa* ssp. rapa has one of the most diversified anthocyanin metabolites in root, which may confer a superior health-promoting attribute. Moreover, comparative analysis of the anthocyanin contents in PT and GT turnip skins in this study showed that they have differential profiles (Figure 4, Table S2) but PT had 20 times more anthocyanin levels than GT, which may explain the difference in their skin coloration.

Variation in anthocyanin content in plants has been linked to the differential expression of key genes encoding structural enzymes involved in the anthocyanin biosynthesis pathways [57,58]. These genes have been classified as early biosynthesis genes and include chalcone synthase (CHS), chalcone isomerase (CHI), and flavanone-3-hydroxylase (F3H), while others are classified as late biosynthesis genes, including dihydroflavonol-4-reductase (DFR), anthocyanidin synthase (ANS), and UDP-flavonoid glucosyl transferase (UFGT) [22]. To uncover the key structural genes modulating the differential pigmentation in skin of PT and GT, we de novo sequenced and assembled the whole transcriptome from skin samples collected at five developmental stages by the RNA-sequencing technology (Table 1, Table 2 and Table 3). Although Lin et al. [43] reported the full sequencing of the genome of *Brassica rapa* ssp. rapa, the sequence is not publicly available and has propelled us for the de novo transcript assembly. A very high number of unigenes was identified in this study (~76,000 genes) as compared to previous reports in *Brassica rapa* genotypes (~40,000 genes) [42,43]. Our results will fuel further investigations on the genetic variation underlying the diverse morphotypes found in this species. Based on the differential gene expression (DEG) analysis and gene annotation, we searched for all DEGs related to the flavonoid-anthocyanin biosynthesis. Our results revealed four DEGs between PT and GT, all classified as late biosynthesis genes. Dihydroflavonols are at the halfway of anthocyanin biosynthesis from the end of the activity of early biosynthesis enzymes and the beginning of the activity of the late biosynthesis enzymes and represent the same substrate for both anthocyanin biosynthesis and flavonols biosynthesis. In various plants, it has been documented that the up-regulation of early biosynthesis genes increases the formation of dihydroflavonols, which later facilitates the high anthocyanin accumulation [30,34,35,37,40]. However, in turnip, we uncovered a different mechanism leading to the differential anthocyanin content (Figure 5 and Figure 6). In fact, the purple turnip (PT) tends to prioritize the anthocyanins biosynthesis over the flavonols biosynthesis by strongly down-regulating one flavonol synthase (*c43941.graph_c0*, FLS) gene, which normally converts dihydroflavonols into flavonols. Then, we predicted that the dihydroflavonols are mainly diverted to the anthocyanins biosynthesis through a strong up-regulation of one DFR gene (*c39842.graph_c0*). This will result in a high accumulation of leucoanthocyanidins in PT. A similar mechanism has been recently discovered in *Mimulus lewisii* [39]. They demonstrated that the gene *LAR1*, encoding an R2R3-MYB transcription factor positively regulates FLS, essentially eliminating anthocyanin biosynthesis in the white region around the corolla throat of *M. lewisii* flowers by diverting dihydroflavonol into flavonol biosynthesis from the anthocyanin pigment pathway [39]. Interestingly, the putative nonsense mutation identified in the coding sequence of the DFR gene could lead to a nonfunctional protein in GT (Figure 7I), which may impair the accumulation of anthocyanins in GT skin. Moreover, PT strongly activated one ANS gene (*c45741.graph_c0*) which will likely generate high level of anthocyanidins. All these genes (DFR, ANS) are essential for the formation of the higher content of anthocyanins in PT but without glucosylation, anthocyanins are unstable and do not accumulate in the cells to give the purple pigmentation [59]. In this regard, we detected one UFGT gene (*c48211.graph_c0*) strongly up-regulated in PT and will likely favor the high accumulation of purple anthocyanin pigments in PT (Figure 5). Analogically, we deduced that the green turnip (GT) prioritizes flavonols biosynthesis through a high activity of the FLS gene and strongly reduces anthocyanin accumulation by down-regulating DFR, ANS and UFGT genes (Figure 6). Nonetheless, it is still unclear how the different anthocyanin profiles were generated in both varieties. For example, how delphinidin and petunidin 3-*O*-glucoside accumulates to higher levels in GT or why pelargonin and pelargonidin 3-*O*-beta-D-glucoside, pelargonidin *O*-acetylhexoside, cyanidin *O*-acetylhexoside are only detected in PT, will require additional investigations.

The activities of the structural genes in the flavonoid-anthocyanin biosynthetic pathways are regulated by other genes, predominantly transcription factors (TF) from the families of MYB, bHLH and WD40, which form ternary complexes called MBW [28,29,31,33]. Accordingly, in this study we also uncovered several members of MYB and bHLH families as the main drivers of transcriptional changes between the two turnips (Figure 3). However, we did not find any annotated gene corresponding to the WD40 within the DEGs. An et al. [32] have shown that the ternary complexes MBW is not indispensable for the regulation of anthocyanin genes in apple, therefore, with pending in-depth investigation, we are tempted to speculate that MYB and bHLH may be sufficient for the regulation of anthocyanin structural genes in turnips. Many WRKY genes were also differentially expressed between the two turnips (Figure 3), suggesting that they may play key roles. Lei et al. [60] demonstrated that *WRKY2* and *WRKY34* negatively regulate the expression of certain MYBs during plant male gametogenesis. Similarly, *AtWRKY40* binds to the W-box in promoters *AtMYB2* to inhibit its expression [61]. Later on, Verweij et al. [38] also showed in two different species how a WRKY gene negatively regulates the complex MYB-bHLH-WD40. In our study, the MYB and bHLH genes were mostly up-regulated while WRKY genes were mainly down-regulated in TP, implying that WRKYs may repress the expression levels of MYB and bHLH members. Among others, we propose four candidate TFs, including *c42189.graph_c1* (WRKY) *c33188.graph_c0* (MYB), *c44079.graph_c0* (MYB) and *c37493.graph_c0* (bHLH) for future thorough functional characterizations in turnip skin pigmentation.

## 4. Materials and Methods

### 4.1. Plant Materials

Two *Brassica rapa* ssp. rapa varieties with different root skin colors (purple turnip ‘PT’ and green turnip ‘GT’) were used as plant materials. We collected healthy and consistent purple and green turnips during five consecutive developmental stages, including seedling stage (S1, 15 days after sowing (DAS)), early stage of fleshy root expansion (S2, 30 DAS), full expansion stage of fleshy root (S3, 45 DAS), maturity stage of fleshy root (S4, 55 DAS) and harvest stage of fleshy root (S5, 65 DAS), at the Anningqu experimental site, Xinjiang, China. Plants were grown in natural environment conditions in July 2017 and skin samples were collected from three different plants of each variety (three biological replicates). In total, 30 samples were quickly frozen in liquid nitrogen and stored at −80 °C in a refrigerator until further use.

### 4.2. Metabolic Profiling

The sample preparation, extract analysis, metabolite identification and quantification were performed at Wuhan MetWare Biotechnology Co., Ltd. (www.metware.cn) following their standard procedures and previously described by Zhang et al. [48]

### 4.3. Metabolite Data Analysis

Before the data analysis, quality control (QC) analysis was conducted to confirm the reliability of the data. The QC sample was prepared by the mixture of sample extracts and inserted into every five samples to monitor the changes in repeated analyses. Data matrices with the intensity of the metabolite features from the 30 samples were uploaded to the Analyst 1.6.1 software (AB SCIEX, Ontario, Canada) for statistical analyses. The supervised multivariate method, partial least squares-discriminant analysis (PLS-DA), was used to maximize the metabolome differences between the pair of samples. The relative importance of each metabolite to the PLS-DA model was checked using the parameter called variable importance in projection (VIP). Metabolites with VIP ≥ 1 and fold change ≥ 2 or fold change ≤ 0.5 were considered as differential metabolites for group discrimination [48].

### 4.4. RNA Extraction, cDNA Library Construction, and Transcriptome Sequencing

Total RNAs were extracted using Spin Column Plant total RNA Purification Kit following the manufacturer′s protocol (Sangon Biotech, Shanghai, China). Purity of the extracted RNAs was assessed on 1% agarose gels followed by NanoPhotometer spectrophotometer (IMPLEN, Los Angeles, CA, USA). RNA quantification was performed using Qubit RNA Assay Kit in Qubit 2.0 Flurometer (Life Technologies, Carlsbad, CA, USA). Next, RNA integrity was checked by the RNA Nano 6000 Assay Kit of the Agilent Bioanalyzer 2100 system (Agilent Technologies, Santa Clara, CA, USA).

Sequencing libraries was created using NEB Next Ultra RNA Library Prep Kit following manufacturer′s instructions. The index codes were added to each sample. Briefly, the mRNA was purified from 3 μg total RNA of each of three replicate using poly-T oligo-attached magnetic beads and then broken into short fragments to synthesize first strand cDNA. The second strand cDNA synthesis was subsequently performed using DNA Polymerase I and RNase H. PCR was carried out with Phusion High Fidelity DNA polymerase using universal PCR primers and index (×) primer. Finally, six paired-end cDNA libraries with an insert size of 300 bp were constructed for transcriptome sequencing and sequenced on Illumina HiSeq 2500 platform (Illumina Inc., San Diego, USA) by Biomarker Technology Corporation (www.biomarker.com.cn). The raw RNAseq data are submitted at: www.ncbi.nlm.nih.gov/bioproject/PRJNA558197.

### 4.5. De novo Assembly, Functional Annotation, Classification and Metabolic Pathway Analysis

The clean reads were retrieved after trimming adapter sequences, removal of low quality (containing > 50% bases with a Phred quality score < 15) and reads with unknown nucleotides (more than 1% ambiguous residues N) using the FastQC tool (http://www.bioinformatics.babraham.ac.uk/projects/fastqc/). The high quality reads from all the 30 libraries were de novo assembled into transcripts using Trinity (Version r20140717, [62]) by employing paired-end method [63]. Next, the transcripts were realigned to construct unigenes. The assembled unigenes were then annotated by searching against various databases such as Kyoto Encyclopedia of Genes and Genomes (KEGG) [64], Gene Ontology (GO) [65], Clusters of Orthologous Groups (COG) [66], PfAM, Swissprot [67], egNOG [68], NR [69], euKaryotic Orthologous Groups (KOG) [70] using BLAST [71] with a threshold of E-value < 1.0 × 10^−5^.

The software KOBAS2.0 [72] was employed to get the unigene KEGG orthology. The analogs of the unigene amino acid sequences were searched against the Pfam database [73] using HMMER tool [74] with a threshold of E-value < 1.0 × 10^−10^. The sequenced reads were compared with the unigene library using Bowtie [75], and the level of expression was estimated in combination with RSEM [76]. The gene expression level was determined according to the fragments per kilobase of exon per million fragments mapped (FPKM).

### 4.6. Differential Expression and Enrichment Analysis

The read count was normalized and EdgeR Bioconductor package [77] was used to determine the differential expression genes (DEGs) between the two varieties at each developmental stage with the fold change > 2 [44] and false discovery rate (FDR) correction set at *p* < 0.01. GO enrichment analysis was performed using the topGO method based on the wallenius noncentral hypergeometric distribution with *p* < 0.05 [78]. KEGG pathway enrichment analysis of the DEGs was done using KOBAS2.0 [72]. The FDR correction was employed (*p* < 0.05) to reduce false positive prediction of enriched GO terms and KEGG pathways.

### 4.7. SNP Analysis

The reads and unigene sequences of each sample were compared using the software STAR [79] and the single nucleotide polymorphism (SNP) was identified through the pipeline (SNP Calling) for RNA-Seq by GATK2 [80]. Raw vcf files were filtered with GATK standard filter method and other parameters (clusterWindowSize: 35; MQ0 ≥ 4 and (MQ0/(1.0*DP)) > 0.1; QUAL < 10; QUAL < 30.0 or QD < 5.0 or HRun > 5), and only SNPs with distance > 5 were retained.

### 4.8. Gene Expression Using Quantitative Real Time-PCR

The qRT-PCR was performed on RNA extracted from root samples of both varieties at the five developmental stages as described by Dossa et al. [81] using the *Actin* gene as the internal control. Specific primer pairs of 15 selected genes were designed using the Primer Premier 5.0 [82] (Table S4). The qRT-PCR was conducted on a Roche Lightcyler^®^ 480 instrument using the SYBR Green Master Mix (Vazyme), according to the manufacturer’s protocol. Each reaction was performed using a 20 μL mixture containing 10 μL of 2 × ChamQ SYBR qPCR Master Mix, 6 μL of nuclease-free water, 1 μL of each primer (10 mM), and 2 μL of 4-fold diluted cDNA. All of the reactions were run in 96-well plates and each cDNA was analyzed in triplicate. The following cycling profile was used: 95 °C for 30 s, followed by 40 cycles of 95 °C/10 s, 60 °C/30 s. Data are presented as relative transcript level based on the 2^−∆∆Ct^ method [83].

## 5. Conclusions

In summary, this study generated tremendous genomic and metabolic resources and elucidated the mechanisms of the differential anthocyanin accumulation in purple and green turnips. It provides an important theoretical basis for further in-depth analysis of the candidate structural genes along with the key transcription factors predicted to modulate anthocyanins in turnip towards developing new turnip varieties with improved nutritional quality.

## Figures and Tables

**Table 1:** Overview of the transcriptome sequencing dataset and quality check.

Samples ID	Read Number	Base Number	GC Content	% ≥ Q30
GT1	29,185,016	8,726,667,568	47.42%	91.63%
GT2	20,717,765	6,196,426,170	47.42%	91.70%
GT3	21,156,763	6,319,382,840	47.27%	91.95%
GT4	27,588,012	8,249,989,480	47.55%	93.05%
GT5	21,971,588	6,574,089,374	47.05%	92.05%
GT6	23,582,547	7,053,356,366	46.92%	91.97%
GT7	37,452,770	11,183,417,788	46.90%	90.76%
GT8	32,252,399	9,647,120,456	47.09%	91.89%
GT9	20,867,338	6,235,980,998	47.36%	90.74%
GT10	32,765,445	9,782,653,688	47.16%	91.39%
GT11	23,201,289	6,937,494,090	47.56%	91.16%
GT12	31,341,050	9,370,066,168	47.21%	91.70%
GT13	20,754,387	6,205,716,562	47.56%	92.23%
GT14	24,312,743	7,274,160,626	47.35%	92.91%
GT15	26,794,514	8,013,487,372	47.48%	92.74%
PT1	24,145,033	7,223,733,604	47.23%	92.31%
PT2	27,681,645	8,273,851,708	47.04%	92.79%
PT3	26,077,522	7,801,143,252	47.51%	92.07%
PT4	25,070,137	7,501,916,476	47.29%	92.08%
PT5	24,021,197	7,187,109,432	47.42%	92.39%
PT6	22,053,479	6,589,934,434	47.30%	92.60%
PT7	24,707,235	7,377,482,668	47.25%	92.60%
PT8	25,937,942	7,760,138,900	47.43%	92.87%
PT9	21,983,093	6,562,135,072	47.19%	91.87%
PT10	28,772,133	8,603,239,086	47.33%	92.59%
PT11	24,124,411	7,219,966,492	47.46%	92.28%
PT12	25,846,725	7,730,236,716	47.35%	92.39%
PT13	26,784,282	8,007,970,564	47.24%	91.47%
PT14	26,748,564	7,995,602,902	47.50%	92.14%
PT15	28,865,986	8,619,275,022	47.07%	92.08%

**Table 2:** Statistics of the unigene assembly results.

Length Range (bp)	Transcript Number	Unigene Number
200–300	35,501 (12.87%)	27,206 (35.73%)
300–500	33,539 (12.15%)	17,833 (23.42%)
500–1000	55,072 (19.96%)	13,519 (17.75%)
1000–2000	93,093 (33.74%)	10,680 (14.02%)
2000+	58,724 (21.28%)	6914 (9.08%)
Total Number	275,929	76,152
Total Length	376,323,528	59,450,389
N50 Length	1921	1443
Mean Length	1363.84	780.68

**Table 3:** Functional annotation statistics of the unigenes.

Annotation Database	Annotated Number	300 ≤ Length < 1000	Length ≥ 1000
COG_Annotation	12,979	4215	6007
GO_Annotation	34,789	13,243	13,960
KEGG_Annotation	17,096	6870	5729
KOG_Annotation	28,073	11,024	9424
Pfam_Annotation	30,231	10,820	13,789
Swissprot_Annotation	29,952	11,517	11,893
eggNOG_Annotation	46,472	18,520	15,908
nr_Annotation	48,834	19,848	16,761
All_Annotated	52,449	21,373	16,967

**Table 4:** Anthocyanins detected in two turnip varieties.

Index	KEGG ID	Compounds	Class
Bra18	-	Peonidin *O*-hexoside	Anthocyanins
Bra23	-	Rosinidin *O*-hexoside	Anthocyanins
Bra1	-	Delphinidin *O*-malonylhexoside	Anthocyanins
Bra8	C08604	Cyanidin 3-*O*-glucoside	Anthocyanins
Bra22	-	Delphinidin *O*-malonyl-malonylhexoside	Anthocyanins
Bra28	C08726	Peonidin	Anthocyanins
Bra32	-	Cyanidin *O*-acetylhexoside	Anthocyanins
Bra35	-	Pelargonidin *O*-acetylhexoside	Anthocyanins
Bra26	-	Procyanidin A1	Proanthocyanidins
Bra29	C10237	Procyanidin A2	Proanthocyanidins
Bra20	-	Procyanidin B2	Proanthocyanidins
Bra34	-	Procyanidin B3	Proanthocyanidins
Bra17	C05908	Delphinidin	Anthocyanins
Bra27	C05904	Pelargonidin	Anthocyanins
Bra5	C12138	Delphinidin 3-*O*-glucoside	Anthocyanins
Bra12	C08620	Cyanidin 3-*O*-rutinoside	Anthocyanins
Bra9	C08718	Malvidin 3,5-diglucoside	Anthocyanins
Bra10	C08725	Pelargonin	Anthocyanins
Bra7	C12139	Petunidin 3-*O*-glucoside	Anthocyanins
Bra11	-	Pelargonidin 3-*O*-beta-D-glucoside	Anthocyanins
Bra25	C05905	Cyanidin	Anthocyanins

## Supplementary Materials

Supplementary materials can be found at https://www.mdpi.com/1422-0067/20/18/4387/s1. Table S1. Statistics of the read mapping results. GP (Green Turnip) and PT (Purple Turnip); Table S2. Quantification of the detected anthocyanins in two turnips. GP (Green Turnip) and PT (Purple Turnip) and S1–S5 represent the different developmental stages. Data from three biological replicates are shown; Table S3. List of the shared and stage-specific differentially accumulated anthocyanins between Green Turnip and Purple Turnip at different developmental stages S1–S5; Table S4. The primer sequences of genes used for quantitative real time PCR.
